# Progress and Challenges in the Development of COVID-19 Vaccines and Current Understanding of SARS-CoV-2- Specific Immune Responses

**DOI:** 10.4014/jmb.2006.06006

**Published:** 2020-06-16

**Authors:** Kyun-Do Kim, Insu Hwang, Keun Bon Ku†, Sumin Lee, Seong-Jun Kim, Chonsaeng Kim

**Affiliations:** Center for Convergent Research of Emerging Virus Infection, Korea Research Institute of Chemical Technology, Daejeon 34114, Republic of Korea

**Keywords:** COVID-19, SARS-CoV-2, Coronavirus, vaccine, immune response

## Abstract

The outbreak of coronavirus disease 2019 (COVID-19) caused by severe acute respiratory syndrome coronavirus 2 (SARS-CoV-2) is spreading globally, and the WHO has declared this outbreak a pandemic. Vaccines are an effective way to prevent the rapid spread of COVID-19. Furthermore, the immune response against SARS-CoV-2 infection needs to be understood for the development of an efficient and safe vaccine. Here, we review the current understanding of vaccine targets and the status of vaccine development for COVID-19. We also describe host immune responses to highly pathogenic human coronaviruses in terms of innate and adaptive immunities.

## Introduction

Coronavirus disease 2019 (COVID-19) is an infectious disease caused by a novel coronavirus called severe acute respiratory syndrome coronavirus 2 (SARS-CoV-2). This virus is a member of the human coronaviruses (HCoVs) and is an enveloped, positive-sense single-stranded RNA virus. Full-length genome sequences of the virus have been obtained, and it shares 79.6% sequence similarity with severe acute respiratory syndrome coronavirus (SARS-CoV) [1]. Four HCoVs that cause mild respiratory illness, HCoV-OC43, HCoV-OC43, HCoV-NL63, and HCoV-HKU1, have been reported [2]. In contrast, two HCoVs, SARS-CoV and Middle East respiratory syndrome coronavirus (MERS-CoV), emerged and caused severe diseases [3-5]. SARS-CoV caused roughly 8000 infections with a 10% mortality rate, whereas MERS-CoV caused over 857 infections with a 35% mortality rate [6]. SARS-CoV-2 is the seventh member of HCoVs and has caused over 60000 infections with a 2.2% mortality rate as of February 13, 2020 [7].

The common symptoms of patients with COVID-19 are fever, cough, or chest tightness, and most patients have reported to experience mild symptoms [8]. Some patients progress to severe disease with dyspnea and pneumonia [9]. The reproductive number (R0) of SARS-CoV-2 was found to be approximately 2.2 based on an estimation using a mathematical model [10]. This indicates that one infected person will pass the virus on to 2.2 people. A comparison of the R0 value of SARS-CoV-2 with those of SARS-CoV and MERS-CoV shows that SARS-CoV-2 is more contagious than the latter [11]. WHO has declared this outbreak a pandemic [12]. Vaccines are effective methods for preventing the transmission of infectious diseases. The immune response of vaccines against SARS- CoV-2 is vital for the control and prevention of this pathogen. However, immunopathogenesis may occur because of an unregulated immune response. Thus, the immune response needs to be understood for efficient and safe vaccine development. This review describes the current status of vaccine development for SARS-CoV-2, and the immune response to SARS-CoV, MERS-CoV, and SARS-CoV-2 infection.

## Development of a SARS-CoV-2 Vaccine

The outbreak caused by a novel coronavirus, SARS-CoV-2, has been spreading to many countries worldwide with high human-to-human transmission [13]. Currently, there is no approved vaccine or therapeutic drug against SARS-CoV-2. There were similar two outbreaks of coronaviruses in the past, one in 2002-2003 by SARS- CoV and the other in 2012 by MERS-CoV [11, 14]. Many efforts have been directed to develop effective vaccines for controlling the spread of the virus [15, 16]. Two Phase I clinical trials on SARS-CoV vaccine and one Phase I clinical trial on MERS-CoV vaccine have been reported [17-19]. According to the reports, the vaccine candidates were safe and could induce the required immune responses. Based on the previous experience of vaccine development against SARS-CoV and MERS-CoV, several research groups and biopharmaceutical companies have been developing prophylactic vaccines. The genetic similarity between SARS-CoV-2 and the two previous coronaviruses strongly supports the fact that the strategies used for vaccine development for the two previous threatening coronaviruses can be exploited to develop a vaccine against SARS-CoV-2 in a timely manner. Potential B-cell and T-cell epitopes for SARS-CoV-2 were screened based on the epitopes for SARS-CoV [20]. Most studies have used the surface-exposed spike protein (S-protein) to induce a response from the host immune system, such as neutralizing antibodies and T-cell response [21]. The S-protein binds to the viral receptor and mediates entry of the virus into the host cells. Full-length S-protein is divided into the S1-subunit responsible for receptor binding and the S2-subunit responsible for cell-membrane fusion [22]. The structure of SARS-CoV-2 spike protein obtained by cryo-electron microscopy was determined in the prefusion conformation [23]. This study provided biophysical and structural evidence that the SARS-CoV-2 S-protein binds at least 10 times more tightly to angiotensin-converting enzyme 2 (ACE2) than that of SARS-CoV. This result suggests that SARS-CoV- 2 also uses the ACE2 as a cell receptor. The receptor binding domain (RBD) is located in the C-terminal domain of the S1-subunit and directly engages with the receptor. The structure of RBD of SARS-CoV-2 with ACE2 was also obtained by cryo-electron microcopy [24]. Vaccines targeting the full-length S, S1 subunit, and RBD have been developed for MERS-CoV and SARS-CoV [15, 16, 25]. Similarly, vaccine candidates for SARS-CoV-2 have been developed targeting this spike protein or its subunits. A wide range of vaccine platforms have been applied to develop vaccines including mRNA vaccines, DNA-based vaccines, recombinant subunit protein vaccines, inactivated vaccines, and viral vector-based vaccines (Table 1). WHO released the draft landscape of COVID-19 candidate vaccines on June 2, 2020, and 10 candidate vaccines in clinical evaluation are summarized in Table 1.

## Innate Immunity

Innate immunity is the first line of defense mechanism of the host immune system against viral infections [26]. Activation of the innate immune response is the initial step for stimulating not only the adaptive immune response but also the systemic immune response against viral infection. After viral invasion, the host immune system recognizes the non-self-antigen of the pathogen using pattern-recognition receptors (PRRs) [27]. Among the several types of PRRs, the toll-like receptor family mainly recognizes viral nucleic acids such as genomic DNA, single-stranded RNA, and double-stranded RNA. For RNA viruses such as coronavirus including SARS-CoV-2, endosomal RNA receptors, TLR3 and TLR7, recognize the viral RNA. This recognition gradually stimulates immune-mediated factors and finally type I interferon (IFN) and inflammatory cytokines that have antiviral effects [28, 29]. In clinical reports on the fatal SARS-CoV or MERS-CoV infection cases, high levels of proinflammatory cytokines caused severe lung damage and viral pathogenesis as the levels of neutrophils and monocyte-macrophage were increased. In addition, high levels of proinflammatory cytokines caused delayed simulation of type I IFN with high viral load in the early stages of infection [30-32]. A similarly deregulated innate immune response is expected in the case of patients with fatal COVID-19. Thus, the innate immune response probably plays a key role in the protective or destructive responses in SARS-CoV-2 infection and needs to be investigated.

### Viral Pathogen-Associated Molecular Patterns (PAMPs)

Recognition of PAMPs is an essential function of the innate immune system. These PAMPs are molecular structures of the pathogen such as glycoproteins, lipopolysaccharides, proteoglycans, and nucleic acid motifs. These microbial foreign antigenic structures are recognized by the host PRRs. The receptors are dominantly expressed by antigen-presenting cells such as dendritic cells (DCs) and macrophages (MPs). The PRRs consists of four types of receptors, Toll-like receptors (TLR), nucleotide-biding oligomerization domain-like receptors (NLR), C-type lectin receptor, and RIG-I like receptor (RLR) [29]. The presence of viral sensing PRRs in various cellular compartments allows the innate immune cells to recognize and quickly respond to a broad range of viruses, which replicate in diverse cells. The TLRs and RLRs are the most common host PRRs that respond to RNA viruses during the entry step of coronaviruses. The S-protein of SARS-CoV-2 binds to the ACE2 of the host cells and induces membrane fusion and release of the viral RNA into the cytoplasm. Endosome associated TLRs, such as TLR3 or TLR7, may detect the viral genomic RNA. However, certain types of coronaviruses have evolved to escape the immunological surveillance system. SARS-CoV and MERS-CoV produce double-membrane vesicles, in which the virus replicates, without exposure of PAMPs [33, 34]. The immune escape mechanism has to be investigated for the newly emerged SARS-CoV-2 to better understand the viral pathogenesis.

### Interferon Response

During the early phases of viral infection, viral replication is hindered by type I IFN, complement proteins, and other innate immune mediators. There are three classes of IFNs: Type I (IFN-alpha and IFN-beta), Type II (IFN- gamma), and Type III (IFN-lambda). Type I IFN is secreted by virus-infected cells and type II IFN is secreted by T cells, natural killer (NK) cells, and macrophages. Type III IFN is not much known about its mechanism and functional effect than other types. The direct antiviral activity of type I IFN is mediated by various different mechanisms, e.g., blockade of viral entry into the target cell, prevention of virion release, and inhibition of viral transcription and translation [35, 36]. In addition to the direct effects, type I IFNs play immunoregulatory roles and are responsible for the critical bridging mechanism between the innate and adaptive immune responses to defend against viral invasion. For example, type I IFNs induce the cytotoxicity of NK cells and enhance the expression of major histocompatibility complex class I protein in most cells and co-stimulatory molecules in antigen-presenting cells [37]. In case of the innate antiviral response against SARS-CoV and MERS-CoV, particularly production of type I IFN, constitutes the first line of defense. Type I IFN mediates antiviral effects by directly inhibiting virus replication and indirectly modulating the host immune response to virus infection, both of which are mediated by the induction of IFN-stimulated genes. Thus, SARS-CoV and MERS-CoV use evolutionary strategies to decrease type I IFN production for survival [38, 39]. Delayed type I IFN signaling orchestrates the inflammatory responses and lung immunopathology in human infection.

The virological character of novel coronavirus, SARS-CoV-2, is distinct when compared with that of original SARS-CoV. Although SARS-CoV-2 has a similar genome component and viral replication kinetics in Vero cell, it is much more sensitive to the pre-treatment by type I IFNs [40]. The high mortality rates in the elderly and nearly zero fatality rate among persons aged less than 19 years suggest that age is an important factor for severe outcome of COVID-19 patients [41]. Age-dependent dysregulation of the innate immune response has been previously reported [42]. Thus, we need to further investigate the relation between the innate immune response and pathogenesis of newly emerged coronavirus.

## Adaptive Immunity

### T Cell-Mediated Immune Response

The immune system can recognize and eliminate viral pathogens. Especially, T cells play a central role in the robust adaptive immune responses against viral infection. CD4^+^ T cells modulate CD8^+^ T-cell responses, humoral immunity, and macrophage-mediated antiviral activity and are involved in recruiting cells to sites of infection. CD8^+^ T cells control viral infections by directly killing infected cells, secreting cytokines, and forming memory that protects against reinfection. Dendritic cells initiate all adaptive immune responses by uptaking, processing, and presenting the viral antigens to activate naive antigen-specific T cells.

For patients infected with SARS-CoV, a significant depletion of T cells in peripheral blood was observed [43]. During the recovery period, the CD8^+^ T-cell count was restored to normal levels within 2-3 months after disease onset. However, the levels of total lymphocytes, CD3^+^ T cells, and CD4^+^ T cells remained significantly lower than the normal levels even one year after disease onset [44]. For patients with COVID-19, CD4^+^ T-cell counts were found to be lower than the normal levels in 45.4% of patients, while the CD4/CD8 ratio was similar to that of healthy controls in 92.8% patients [45]. Recently, another research group also reported that the number of T cells was significantly lower than normal levels in patients with COVID-19. They also found that patients with COVID-19 exhibit markedly reduced levels of regulatory T cells, especially in severe cases [46]. Patients with severe COVID-19 symptoms exhibited increased expression of regulatory molecules and decreased expression of functional cytokines in the peripheral blood. In particular, the levels of IFN-γ and TNF-α in CD4^+^ T cells were decreased in patients with severe symptoms than in patients with mild symptoms, whereas the levels of granzyme B and perforin in CD8^+^ T cells were increased in patients with severe symptoms than in patients with mild symptoms. These results collectively indicate that the imbalance in T-cell subsets may negatively regulate host antiviral adaptive immunity [47].

CD4^+^ T helper cells express proinflammatory cytokines to protect the host cells against viruses. However, SARS-CoV induces T-cell apoptosis to inhibit the T-cell response. In a mouse model, rapid SARS-CoV induced delayed IFN-α/β response which coincided with an immoderate influx of pathogenic inflammatory monocyte- macrophages (IMMs). The delayed IFN signaling also impeded virus clearance by IMM responses and T-cell apoptosis [48].

In response to virus infections, our immune systems predominantly establish Th1-biased immunity leading to sustained CD8^+^ T-cell responses to kill virus-infected cells [49]. Th1 cytokine, IFN-γ, and proinflammatory cytokines, IL-1β, IL-6, and IL-12, are elevated in the serum of SARS patients [50]. The cytokine profile of MERS patients also showed marked elevation of proinflammatory cytokines, IFN-γ, TNF-α, IL-15, and IL-17 [51]. In response to SARS-CoV-2 infection, a significant elevation in the concentrations of IL1β, IFN-γ, IP10, and macrophage chemotactic protein 1 (MCP-1) was observed, probably leading to activated Th1-cell responses. Unlike SARS-CoV and MERS-CoV, SARS-CoV-2 infection also elevated levels of Th2 cytokines, IL-4 and IL-10. Because the balance of Th1 and Th2 cells is critical for antiviral immunity, future studies need to clarify the roles of Th1 and Th2 on antiviral immunity throughout SARS-CoV-2 infection [52].

Activated CD8^+^ T cells express activation markers, CD38 and HLA-DR, following viral infection [53]. Similar to previous research on the Ebola and influenza viruses, the frequency of CD38+HLA-DR+ CD8^+^ T cells was found to have promptly increased in patients with COVID-19 from day 7 (3.57%) to day 8 (5.32%) and day 9 (11.8%) and then decreased at day 20 (7.05%). Moreover, patients with COVID-19 showed much higher frequency of CD38+HLA-DR+ CD8^+^ T cells than healthy controls (1.47% ± 0.50%; *n* = 5). CD38+HLA-DR+ CD4^+^ or CD8^+^ T cells expressed higher level of granzymes A and B and perforin than ungated CD4^+^ or CD8^+^ T cells [54].

Cytokine storm is a systemic inflammatory response characterized by the uncontrolled proliferation or activation of T cells, B cells, NK cells, macrophages, and monocytes and an associated overproduction of inflammatory cytokines [55, 56].

In some SARS or MERS patients, virus infection triggered cytokine storm, thereby causing severe lung injury. New evidence shows that patients with severe COVID-19 exhibited signs of cytokine storm. Therefore, treatment of cytokine storm would be the key to the survival of patients with COVID-19.

### Antibody Mediated Immune Responses

Humoral immunity mainly involves complement proteins, antimicrobial peptides, and antibodies. Among these, the antibody mediated immune response plays an essential role against coronavirus (CoV) infection. CoV accumulates B lymphocyte subsets [57, 58]. Moreover, potent humoral immune responses to SARS-CoV were observed in SARS-CoV infected patients [59, 60].

Neutralizing antibodies that can block virus entry into the host cells are very important for neutralizing viral infectivity. Both SARS-CoV and MERS-CoV use their S proteins to bind to their host cell receptors, ACE2 and dipeptidyl peptidase 4, respectively [61, 62]. Therefore, the S proteins in their envelopes are useful targets for neutralizing antibody production because these antibodies may be able to block virus entry into host cells. A previous study has also shown that S proteins can stimulate neutralizing antibody production as major antigenic proteins [63].

Antibody responses against CoV infection peaked in the convalescent patient sera and then decreased after recovery. In case of MERS-CoV infection, antibody responses were first detected at days 14-21 after infection, and increased antibody concentration was maintained until more than 18 months of infection [62]. Indirect enzyme- linked immunosorbent assay, which uses the anti-MERS-CoV nucleocapsid antibody, showed that 86% of human serum samples were shown to contain neutralizing antibodies at 34 months of infection, and the titers were the same as those after 13 months of infection. In contrast, 29% human serum samples showed lowered titers of neutralizing antibodies after 34 months of infection [64]. In case of SARS-CoV infection, antibody responses are detectable as early as day 4 after disease onset, and most patients exhibited these antibody responses by day 14. The anti-SARS-CoV antibody responses are detectable for up to 24 months of infection; these responses completely disappeared after 6 years of infection [65, 66]. Similar to anti-SARS-CoV antibody responses, anti-SARS-CoV-2 IgG was first detected at day 4 after disease onset and peaked 4 weeks later, whereas IgM was first detected at day 3 after disease onset and peaked 3 weeks later [67]. In addition, the first COVID-19 patient in Finland also showed a similar anti-SARS-CoV-2 antibody response profile. Anti-SARS-CoV-2 IgG and IgM, which include neutralizing antibodies, were detected within 9 days; these antibodies mostly recognized nucleocapsid and spike proteins [68].

Severe cases of SARS-CoV infection are associated with early seroconversion and higher antibody levels. In contrast, lower antibody titers were observed in mild cases of SARS-CoV infection. Although anti-SARS-CoV antibody mediated immune responses aim to limit virus infection, they also promote the secretion of proinflammatory cytokines, including MCP-1 and interleukin-8 (IL-8). It maybe induce the fatal acute lung injury in SARS-CoV-infected patients [69]. Similar to SARS-CoV infection, SARS-CoV-2 infection is associated with severe symptoms as well as higher IgM and IgG levels, which induce high IFN-γ, IL-6, TNF-α, and IL-10 secretion [67].

CoV infection induces proliferation of memory B cells that can differentiate into plasma cells. Thus, when humans are re-infected with the same or similar epitopes containing CoV, the antibody mediated immune response may react immediately and thereby protect humans from CoV infection [11, 58].

## Summary

COVID-19 has been spreading exponentially globally. There is an urgent need for rapidly developing vaccines to prevent the spread of SARS-CoV-2. Many vaccine candidates are currently being developed using various technologies. Most of these approaches use the S-protein of SARS-CoV-2 as a target. Information on this vaccine has been gained to develop more efficient and safer vaccines. The current vaccine development status is summarized in Table 1.

**Fig. 1:**
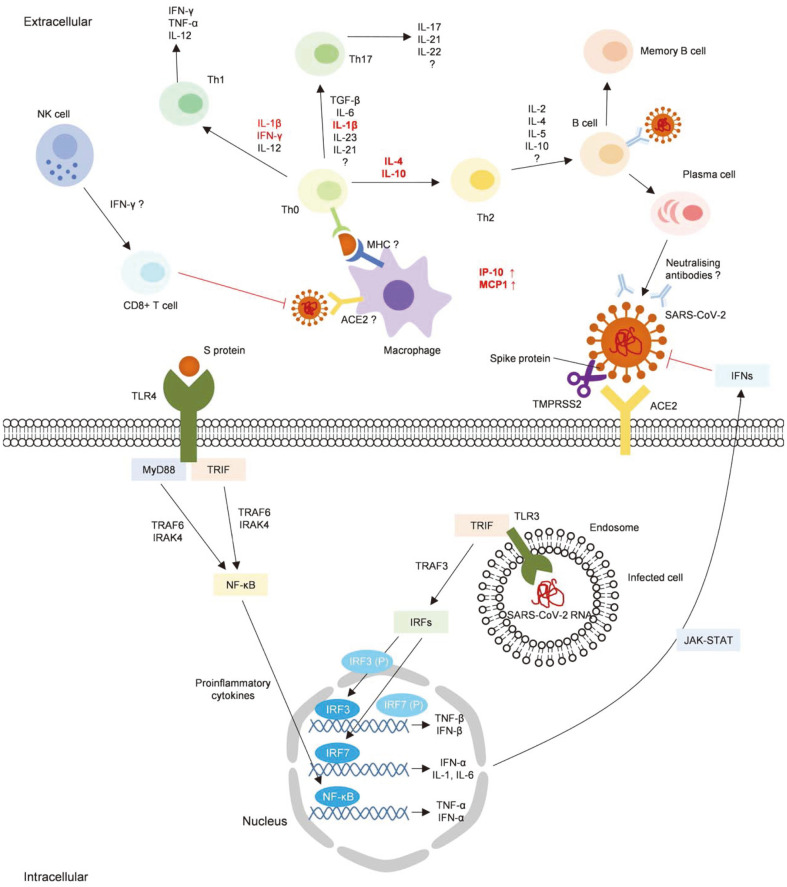
The innate and adaptive immune responses to COVID-19. Immune responses to human coronaviruses are summarized. Newly reported immune response to SARS-CoV-2 is highlighted in red.

Because of the similarity between SARS-CoV-2 and the two previous highly pathogenic human CoVs (SARS- CoV and MERS-CoV), previous researches on immune response to SARS-CoV and MERS-CoV may help understand the immune response to SARS-CoV-2 and develop a safe and effective vaccine. The innate and adaptive immune responses against human coronavirus infection are summarized based on previous reports in Fig. 1 [58, 62].

## Figures and Tables

**Table 1:** Vaccine candidates against SARS-CoV-2 infection and the stages of their clinical tests.

Developer	Platform	Type of candidate vaccine	Current stage of clinical test
University of Oxford/AstraZeneca	Non-replicating viral vector	ChAdOx1-S	Phase 2b/3 Phase 1/2
CanSino Biological Inc./Beijing Institute of Biotechnology	Non-replicating viral vector	Adenovirus Type 5 vector	Phase 2 Phase 1
Moderna/NIAID	RNA	LNP-encapsulated mRNA	Phase 2 Phase 1
Wuhan Institute of Biological Product/Sinopharm	Inactivated	Inactivated	Phase 1/2
Beijing Institute of Biological Product/Sinopharm	Inactivated	Inactivated	Phase 1/2
Sinovac	Inactivated	Inactivated + alum	Phase 1/2
Novavax	Protein subunit	Full length recombinant SARS CoV-2 glycoprotein nanoparticle vaccine adjuvanted with Matrix M	Phase 1/2
BioNTech/Fosun Pharma/Pfizer	RNA	3 LNP-mRNAs	Phase 1/2
Institute of Medical Biology, Chinese Academy of Medical Sciences	Inactivated	Inactivated	Phase 1
Inovio Pharmaceuticals	DNA	DNA plasmid vaccine with electroporation	Phase 1
